# On Constitutional Syphilis

**Published:** 1849-01

**Authors:** G. L. Cooper

**Affiliations:** Bloomsbury Dispensary


*On Constitutional Syphilis.* By Mr. G. L. Cooper, of the Bloomsbury Dispensary.—Syphilis, in its secondary forms, or constitutionally considered, is the result of, or consequent upon, the absorption of a morbid poison into the circulation, reappearing in certain symptoms and parts, according to the stage of the disease. Its frequency every practical surgeon must admit, but the cause remains an undecided question. According to the opinions of some writers of indisputable reputation, its frequency is, in the majority, after the treatment of primary syphilis by a mercurial course; others, again, deny this point, on the principle of a non-eradication of the virus by an omission of that mineral. Now, according to my own experience, I most decidedly lean towards the latter, feeling persuaded that mercury is very requisite to accomplish a permanent cure of the true Hunterian chancre. Most surgeons are aware that ulcers of every character which may appear on the penis, or on any other part of the body, will oftentimes heal of their own accord, without any treatment whatever; but it is another thing to suppose the disease has been removed from the system; in numerous instances, both in private and public practice, the merits of these two methods of treatment have been tested by me, and I have found by observation that secondary symptoms do not so frequently follow a judicious course of mercury as when mere alteratives have been administered, neither are they increased in severity on appearing after a mercurial treatment. I am aware this statement has often been made, but it is not in accordance with my results, for these circumstances I should attribute either to an abuse, or to a neglect, of the necessary precautions whilst the patient is under its influence. A well-marked case, treated by me on the non-mercurial system, a short time ago came under my care, and as it bears well upon that subject, I shall select it out of many others. A gentleman, an artist by profession, married, consulted me for a chancre on the prepuce, which, according to his statement, he had contracted from a woman who had sat for one of a group of nymphs; being overcome by her charms in a state of nudity, he was induced to overstep the path of rectitude, and, in the course of ten days after, discovered, much to his chagrin, this legacy of his amour. Being very desirous of avoiding suspicion in the mind of his wife, he particularly requested the iodide of potassium to be prescribed, fearing the mercurial odour might betray him. I accordingly conceded to his wish on his own responsibility; aided by a zinc lotion, the chancre speedily cicatrized. In the course of two months afterwards I was summoned to attend him for a deep excavated ulcer on the right tonsil, with copper-coloured scaly blotches over his body, arms, and face. Considering himself to be free from any chance of suspicion, he readily adopted whatever was recommended; accordingly, I put him under a gentle ptyalism, with rigid restrictions as to diet, and exposure to cold or damp. In a few weeks he was quite recovered, and attached much blame to his own folly. With reference to this subject, I consider the remarks made by Mr. B. Bell to be very just: he says that "a chancre might frequently be cured with external applications alone, and as we know from experience that the virus is not always absorbed, the cure would in a few instances prove permanent: but as we can never with certainty know whether this would happen or not, while in a great proportion of cases there would be reason to think that absorption would take place, we ought not in any case to trust to it." The reports which have issued from the army surgeons on the non-mercurial treatment of syphilis are undoubtedly most interesting, but daily experience convinces me that all ulcers appearing on the genitals are not of a syphilitic character, consequently not liable to be followed by any secondary symptoms; excoriations, herpetic eruptions, and even small ulcers, are frequently witnessed on these parts, often difficult to be distinguished from a true venereal sore; but these readily yield to a simple treatment, being merely the result of a depraved secretion.—*London Lancet.*

